# The role of knowledge management and sharing in cooperatives practices toward National Economic Recovery in the COVID-19 pandemic era

**DOI:** 10.3389/fpubh.2022.963755

**Published:** 2022-12-06

**Authors:** Nur Khasanah, Jaka Sriyana, Andjar Prasetyo, Vita Nurdinawati, Agustinus Hartopo, Heri Wahyudianto, Dewi Gartika, Mochammad Fahlevi

**Affiliations:** ^1^Doctoral Program in Economics, Universitas Islam Indonesia, Yogyakarta, Indonesia; ^2^Department of Economics, Universitas Islam Indonesia, Yogyakarta, Indonesia; ^3^Regional Development Planning Agency, Magelang, Central Java, Indonesia; ^4^Jurusan Teknik Elektromedik, Poltekkes Kemenkes Jakarta II, Jakarta, Indonesia; ^5^Regional Development Planning Agency of Papua Province, Jayapura, Indonesia; ^6^Research and Development Agency of West Java Province, Bandung, Indonesia; ^7^Management Department, BINUS Online Learning, Bina Nusantara University, Jakarta, Indonesia

**Keywords:** National Economic Recovery, cooperatives, knowledge management, policy sharing, COVID-19 pandemic

## Abstract

The National Economic Recovery (NER) Program is one of the responses initiated by the government in Indonesia's economic recovery due to the impact of COVID-19, the target is to reduce the activities of affected communities, including cooperatives. One of the priority aspects for the program to run well and smoothly is the role of institutions in knowledge management and process sharing. This paper examines the role of knowledge management and sharing in cooperatives with qualitative limitations at the knowledge process level, knowledge design level, strategic interaction level, social participation level, academic and scientific ecosystem level, and network and partnership level. A qualitative description becomes a research method with secondary data in the form of a comparison of cooperatives in 2019–2021 as a representation before and during the COVID-19 pandemic. COVID-19 secondary data for 20 months from April 2020 to September 2022 in Indonesia dynamically also support sharpening the analysis. The source of cooperative data is from the publications of the Ministry of Cooperatives and SMEs, while the source of COVID-19 data comes from the publication of the COVID-19 Task Force. The analysis is carried out by building qualitative aspects into quantitative ones that can be formulated in the form of cooperative applications. The result is that the application of the knowledge process level, knowledge design level, strategic interaction level, social participation level, academic and scientific ecosystem level, and network and partnership level can improve decision-making, capture, share, and measure institutional knowledge for the success of the NER Program.

## Introduction

The pandemic of COVID-19 has disproportionately affected businesses worldwide (1). The Indonesian government had considered increasing the role of SMEs in exports, but the current pandemic threatens many of these SMEs' ability to survive. The Indonesian economy has been significantly impacted by this lockdown process, which began with the restriction of non-essential economic activity, and the announcement of the nationwide lockdown in 2020 had a particularly negative impact on SME owners and employers. Indonesian businesses faced more difficulties because of ongoing issues including industrial and global development. The National Economic Recovery (NER) Program is one of the responses initiated by the government to Indonesia's economic recovery due to the impact of COVID-19. This program is based on the Government Regulation of the Republic of Indonesia Number 43 of 2020, although there are already models of incentive schemes that develop incentive mechanisms between the government and private investors from a behavioral preference perspective (2). NER is a government effort to promote SMEs by intervening in interest/margin subsidies; Expenditures on Guarantee Service Fees (IJP); Placement of Government Funds in banking; Guaranteed SMEs credit limit loss; the final income tax for SMEs is borne by the government; Investment financing for cooperatives through the Revolving Fund Management Agency (called LPDB) for SMEs cooperatives; and the Presidential Assistance Program (called Banpres) for Productive Micro Enterprises. This program is a scheme that is expected to be able to develop the joints of the Indonesian economy. The SMEs industries are the major drivers of the Indonesian Economy, significantly contributing to the Gross Domestic Product (GDP) of the nation.

Cooperatives are used as one of the target objects for NER, which targets external and internal issues such as the regulatory framework, the origin of cooperatives, the diffusion of financial cooperatives, network arrangements, business models, banking relations, balancing the interests of members, tax treatment, efficiency, sustainability, mergers, acquisitions, failures, the benefits (and challenges) of FinTech, and the financial contribution of cooperatives to the real economy, including in times of crisis, are interesting to study, which is shown by several scientific contributions, such as the identification result of Kyazze et al. (3) that finds predictors of social performance in cooperatives society from the perspective of developing countries, also explains the concern for cooperatives. Two factors in Yuliarmi et al. (4) research, namely social capital and cooperative empowerment have a positive and significant effect on the welfare of the people who are members of cooperatives and social capital can strengthen the positive influence of cooperative empowerment on community welfare. Then, McKillop et al. (5) reveal the important factors in cooperatives, namely, first, the structural and behavioral characteristics of financial cooperatives, and second, the performance and contribution to the real economy. According to Fernandez-Guadaño et al. (6), cooperatives adjust wages down rather than lay off workers during a recession, then cooperatives' tax contributions to the state are lower because they are subject to a more favorable tax system. In the field of Agriculture, the contribution of Candemir et al. (7) discovers how cooperatives play an indispensable role in the sustainability of the agricultural economy and the adoption of environmentally friendly practices, demonstrating that public policies and private initiatives in cooperatives can be complementary.

The comparison of cooperatives was also revealed by Yakar Pritchard and Çaliyurt (8) with his finding that the economic performance indicator of the level of disclosure of cooperatives engaged in the financial services sector is higher compared to cooperatives engaged in other sectors. The conclusion from Billiet et al. (9) shows the strength of cooperatives in the COVID-19 period with the conclusion that cooperatives are hybrid organizations that maximize value, and not profit. They are owned, regulated, and controlled by their members. Cooperatives are more resilient than conventional companies in times of crisis, due to their special governance characteristics that ensure member centrality. Next to member centrality, cooperative engagement in the local and global environment of the movement enhances the centrality of the mission as well as trust and solidarity among its members, local communities, and other cooperatives. Further advances from theoretical and empirical insights into the relationship between cooperatives and social capital, placing particular emphasis on rural and agricultural cooperatives, which are reviewed by Saz-Gil et al. (10), have also strengthened the function of cooperatives in society.

Meanwhile in Indonesia, in the era of the COVID-19 pandemic quantitatively, according to data from the Ministry of Cooperatives and SMEs of the Republic of Indonesia, active cooperatives have increased from 123,048 cooperatives in 2019, in 2020 as many as 127,124 cooperatives, and in 2021 reaching 127,846 cooperatives. However, in practice, the quantity of cooperatives has not been directly proportional to the quality of cooperatives, although, in 2019–2021, it showed an increase in the quality of cooperatives which was marked by the ownership of a certificate of the cooperative registration number. Certificate of Cooperative Identification Number which is equipped with a QR Code, group type and business scale, and cooperative ranking.

Certificates are given by the government to cooperatives as an appreciation and are recognized as institutionally and business active cooperatives. The low number of cooperatives that have a cooperative registration number certificate has an impact on the slow recovery of the national economy. In addition, even though they already have a cooperative registration number certificate, they also need to maintain the performance of cooperatives with management that can synergize and collaborate toward NER. In an average of three years during the COVID-19 pandemic, only 25.45% of cooperatives had a certificate of cooperative registration number. Therefore, efforts are needed to increase the acceleration of the quality of cooperatives, which can be done through a knowledge management approach and sharing about cooperatives. A model that is used to provide and disseminate knowledge (11–13), ideas, experiences, or skills from a person, department, organization, agency, or company to create a basic need for cooperation. This paper examines the role of knowledge management and sharing in cooperatives with qualitative limitations at the knowledge process level, knowledge design level, strategic interaction level, social participation level, academic and scientific ecosystem level, and network and partnership level.

## Methods

The method used in this research is qualitative research, which includes the use of non-statistical data to describe or explore certain phenomena. The method, which begins with how to emphasize organizing, coordinating, and synthesizing large amounts of data, in this case, cooperatives and COVID-19, is then narrated subjectively inductively on data and value content. In the next stage, developing values and drawing conclusions based on data, oriented to complex processes and rich experience (containing), regardless of numerical data. Several characteristics were used in this study, including using procedures to get the right data; limiting the research to the assumptions and characteristics of the qualitative approach; using a qualitative approach in his research; starting research with a single focus; containing detailed methods, appropriate approaches in data collection, data analysis, and report writing, and analyze the data using a split analysis in several levels. Secondary data in the form of a comparison of cooperatives in 2019–2021 as a representation before and during the COVID-19 pandemic. COVID-19 secondary data for 20 months from April 2020 to September 2022 in Indonesia dynamically also becomes support in sharpening the analysis. The cooperative data source is from the publications of the Ministry of Cooperatives and SMEs, while the COVID-19 data source comes from the publication of the COVID-19 Task Force. The analysis is carried out by building qualitative aspects into quantitative aspects of knowledge processes, knowledge design aspects, strategic interaction aspects, social participation aspects, academic and scientific ecosystem aspects, and network aspects.

## Results and discussion

### Indonesian cooperatives in the COVID-19 pandemic

**Figure 1:**
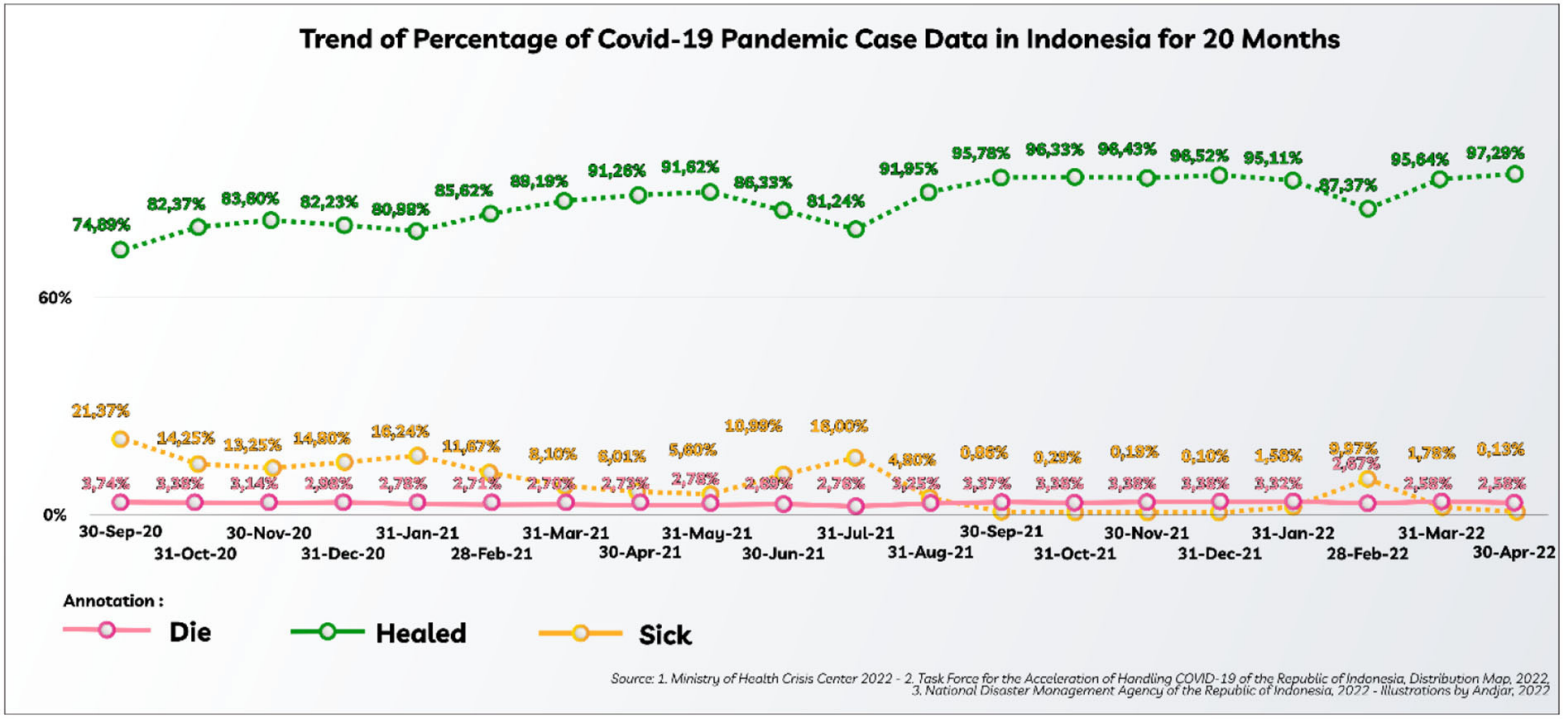
The trend of percentage of COVID-19 pandemic case data in Indonesia for 20 months.

COVI D-19 can paralyze the joints of the Indonesian and global economies due to the large number of victims who have been confirmed positive, have died, and are sick. In the publication of the Ministry of Health of the Republic of Indonesia dynamically for 20 months from 20 April 2020 to 30 September 2022, there is an average population of 7,949 confirmed positive people, and the average population dying every day is 205 people. Although the cure rate is also almost the same as the number of confirmed positives, as many as 7,736 people, which can be explained in Figure 1.

Figure 1 explains that on 30 April 2022, there were 6,046,796 positive (confirmed) cases, consisting of 156,257 dead, 5,882,660 recovered, and 7,879 still sick. The conditions on 30 April 2022, jumped sharply when compared to 30 September 2020, there were 287,008 positive (confirmed) cases, consisting of 10,740 people who died, 214,947 people who recovered, and 61,321 people who are still sick. Although in terms of quantity, it shows that more victims have been identified, the percentage has decreased. The victims of the COVID-19 pandemic have also paralyzed economic activity with an extraordinary impact. Therefore, efforts were made to recover the economy as part of increasing economic activity.

Cooperatives as one of the components that drive the economy in Indonesia have an important portion considering the government's decision to make cooperatives a component of NER.

**Figure 2:**
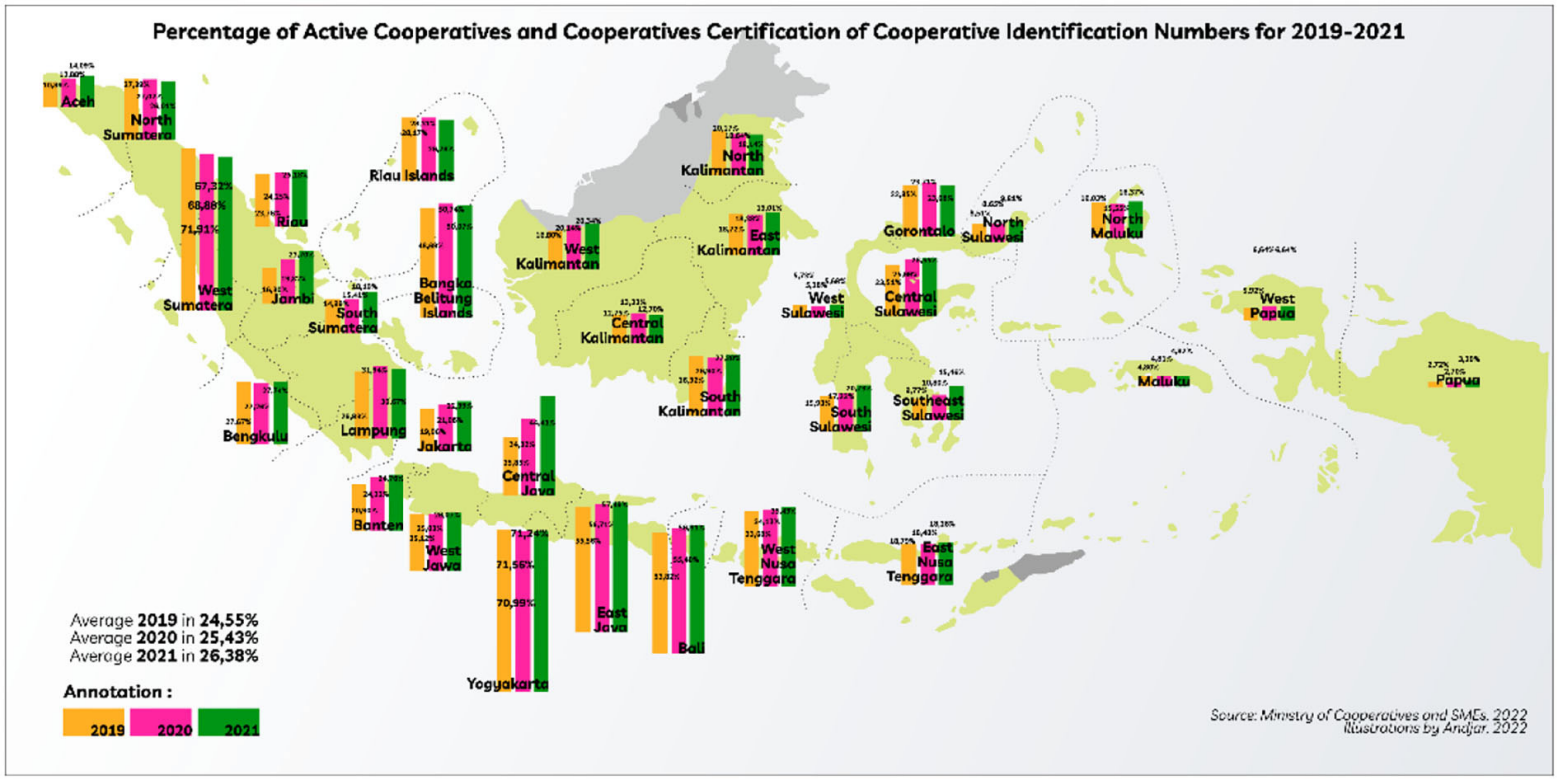
Percentage of active cooperatives and cooperatives certification of cooperative identification numbers for 2019–2021.

In the COVID-19 pandemic during the 2019–2021 period (Figure 2), it was recorded that in 2019 at least as many as 123,048 active cooperative units, only 35,761 cooperative units, or an average of only 24.55% who already have a Cooperative Identification Number Certificate. Then in 2020, it reached 127,124 cooperative units, and only 38,865 cooperative units or an average of 25.43% already had a Cooperative Identification Number Certificate. In 2021, it shows an increase compared to 2020, with an average of 26.38% coming from 127,846 active cooperative units and 41,231 cooperative units that already have a Certificate Number of Cooperatives. The use of bridging organizations increases cooperation between all parties involved (14). The performance of cooperatives as an institution has various measures as indicated by aspects, dimensions, and indicators. In this paper, the aspects discussed consist of the knowledge process aspect, knowledge design aspects, aspects of strategic interaction, aspects of social participation, aspects of the academic and scientific ecosystem, and network aspects.

**Figure 3:**
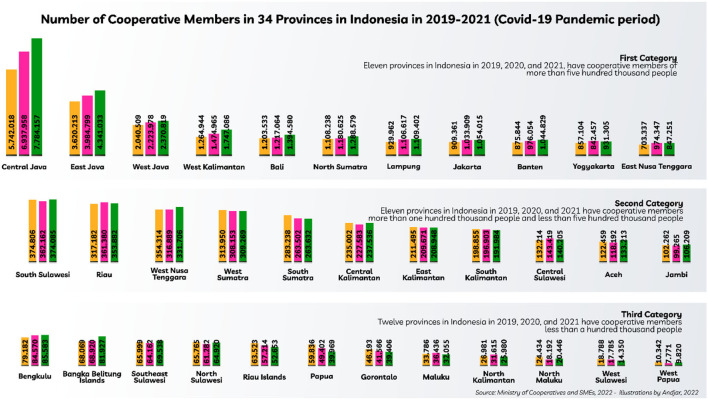
Total of cooperative members in 34 provinces in Indonesia in 2019–2021 (COVID-19 pandemic period).

Indonesian cooperatives within the limits of the number of members nationally in the 2019–2021 period have increased with details in 2019 as many as 22,463,738 people, then in 2020 as many as 25,098,807 people, and in 2021 as many as 27,100,372 people. However, not all the number of members in each province is in line with the number at the national level. This paper is categorized into three scales, provinces that have more than one million members, provinces that have <1 million members and more than five hundred thousand members, and provinces that have cooperative members below five hundred thousand (refer to Figure 3).

In the first category, there are five provinces in a row that have the largest number of cooperative members, then alternately when compared to 2019–2021. Furthermore, in the second category there are differences in the number of provinces that have members between 2019 and 2021, the same thing also happens in the third category. In the introduction, it has been stated that the number of cooperatives equipped with the presentation of data in the discussion describes that the readiness of cooperatives in the context of acting as one of the actors of NER is still not ready. One of the unpreparedness of cooperatives is knowledge management about cooperatives. In the discussion that follows, several management substances are described that can increase the role of cooperatives in actively participating in supporting NER.

### Knowledge management and sharing in cooperatives

Several kinds of literature have raised knowledge management and sharing, which shows that this issue is important to be discussed even though the substance of the study is different (15), thus finding that customer knowledge sharing is an active relationship management process that relies on the factors of customer dedication-based motivation vs. customer concerns. Knowledge sharing is found to be positively linked with cost reduction, enhanced efficiency, improved performance of both the employees and the entire organization, and fostered teamwork within the organization (16). Afterward, Demir et al. (17) concluded that knowledge sharing had a significant impact on knowledge utilization, ability, motivation, and opportunity to practice sustainable development are influenced by knowledge sharing (18). Total quality management practices with their dimensions affected employee performance through knowledge sharing (19). Studies by Imran et al. (20) reveal that management branch banks can improve the effectiveness of their knowledge management in terms of innovation, efficiency, and adaptability through the mediating role of knowledge-sharing processes. Leveraging knowledge management (KM) processes such as knowledge sharing is vital in determining the performance of organizations (21). Knowledge Process (KP) is an initial stage that is intended to improve decision-making, capture, share, and measure cooperative knowledge through KM methodologies and mechanisms. The KP aspect is characterized by the following parameters: There are policies and programs for the sustainable implementation of cooperatives; There are Standard Operating Procedures for implementing cooperatives in various parts; There is a cooperative taxonomy that is agreed upon by members and has been classified as a cooperative document; Use of KM metrics about cooperatives; Transfer of knowledge from experienced staff to new staff about cooperatives; Cooperative knowledge is regularly shared; There are coaching and mentoring programs for cooperative management and members; There are clear methods of documentation of cooperative activities for Preservation of cooperative documents, Open access/open sources to certain data, Research for cooperatives, Access to information, periodic reporting, Mentoring, Meeting reports, results of Ecosystem practices, Lessons learned, Sharing and managing Important Information and Social Networks.

The role of KM and sharing the limitations of qualitative collaboration on these six aspects is based on several scientific literacy references. Process aspects of Knowledge and sharing refer to the KP which was supported by the role of the platform (22), it is also explained that the formal KP affects structural capital but is not significant in the development of human and relational capital which is an interesting finding from (23). Furthermore, it is explained that knowledge sharing has a positive effect on teacher literacy skills (24). Then, it is complemented by the results of a paper which states that knowledge sharing is a key factor in knowledge-based innovation and a stimulating task for management disciplines worldwide during the COVID-19 pandemic (25), corroborated by the statement that knowledge sharing is a part of the KMP (26). Finally, it was concluded that knowledge sharing can occur directly between knowledge providers and knowledge seekers if knowledge is not retained in some form that is easy to find and retrievable (27). To maintain the continuity of cooperative activities, it is directed gradually by encouraging acceleration at each stage to be achieved, these stages can be described as the basic stage, the cooperative has shared knowledge but is *ad hoc* and the knowledge of the cooperative lies with key individuals rather than in an iterative process documented in the description policies, programs, job descriptions, and SOPs. This basic stage generally applies to active and or inactive cooperatives. The second stage is characterized by the existence of some basic KM mechanisms and processes (e.g., formal meeting notes, trip reports, SOPs, documentation, etc.) but they are not always accessible and updated and are not required in policy or practice. Then, in the third stage, the cooperative has many defined KM processes (lessons, trip reports, mentoring, mentoring, etc.) that are guided by formal policies and procedures. In the fourth stage, the cooperative has a formal basic knowledge-sharing strategy within the cooperative. The fifth stage provides an ideal description of cooperatives because KM sharing is integrated into business processes, job descriptions, and institutional functions. The metrics are used to quantitatively measure the cooperative's KM processes and capacities, and continuously improve the cooperative's performance. The management of the cooperative and its multisectoral partners is a shared learning organization to create an institutional culture that encourages the flow of knowledge throughout the organization through KM processes, tools, and technology.

The second aspect is described in Knowledge Design, showing the benefits that Knowledge architecture (KA) establishes the basic foundation for the successful implementation of a short or long-term KM program (28). The workflow-integrated architecture disintegrates the knowledge base, provides a lower collaboration potential, and may require high management efforts, while a workflow-decomposed architecture makes project management easy but provides little added value from the inter-organizational setting (29). The importance of design knowledge conveyed by Oliver et al. (30) is that hundreds of cities and local and national governments are struggling to develop transformational policies but lack the appropriate KA to inform their decision-making. van Gent et al. (31) mentioned that KA provides a general conceptual framework suitable for the development of any complex product. Moscoso-Zea et al. (32) mentioned that the creation of a KA can visualize the current situation and make decisions for the implementation of a new project or initiative. Architectural knowledge and information capital bring about theoretical innovation and assist stakeholders in understanding and influencing strategic learning (33). Knowledge Design emphasized technical practices for policy and KM and sharing, processes, infrastructure, tools used, and how to strengthen skills that are part of the cooperative organizational framework. This technique is characterized by the existence of management and knowledge sharing that has been integrated into cooperative business processes, cooperative job descriptions, institutional functions on the competence of cooperative HR, and KM skills of cooperative technical staff which is indicated by the existence of an agenda/curriculum to train staff about cooperatives: Use of information scientific research for decision making related to the development of cooperatives, and cooperative productivity. The components needed to support this aspect can be provided access. Utilization and evaluation of information technology is needed to support cooperative priorities, communication between members, and most importantly as a tool that facilitates effective knowledge collaboration. Update on the development of cooperatives in terms of methodology/process/policy as part of the process of facilitating public access to the cooperative performance.

The basic stage of maturity of this aspect is marked by the presence of KM is felt like a necessity, but there is little knowledge and expertise in cooperative management. The achievement of cooperative performance in the second stage can be seen by efforts to utilize some of the basic KM technologies and tools available, but cooperatives are not consistent or organized, and accessing institutional knowledge is time-consuming and difficult. The third stage will be seen when the cooperative has had awareness among the leadership and staff about the key concepts and importance of cooperative KM. The fourth stage of this aspect is reached when strengthening KM skills is part of the training program. A formal KM framework has been established within the cooperative, with strong policies, processes, and mechanisms for KM and knowledge sharing both internally and externally to the cooperative. The fifth stage is reached when the KM system is fully operational which is characterized by technology integration with a clear content design.

The aspect of Strategic Interaction is one of the important aspects mentioned by Seo et al. (34) The quality of strategic communication can be an important factor in achieving a competitive advantage and realizing a differentiation strategy. Even confirmed by (35), strategic communication is an economic alternative, which allows developing nations and countries under pressure from Western political, financial, and economic institutions to remain engaged in global economic processes. More technical aspects are discussed by Palmieri and Mazzali-Lurati (36), which are the importance of strategic communication focusing on the analysis, strategic assessment of messages, and the dynamics of strategic communication at the micro level (37). Communication design, participatory communication with community involvement, evidence-based advocacy, and preparedness for risk communication are required for effective communication and health and development. Furthermore, it has also been discussed how it relates to other things (38), identifying the relationships between strategic communication, knowledge co-production, and power, which enables the development of strategic collaborative practices. Developing strategic communication courses has become a trend in educational institutions (39); in economics, it is stated that it provides financial advisers with practical perspectives and guidance on how to communicate effectively in private (40). Strategic interactions, strategic tools, and methodologies to support decision making require the involvement of all parties in creating cooperative sustainability. Community interaction strategy in the context of cooperatives is linked to priority institutional issues and encourages change (individual, social, and political) that leads to the achievement and maintenance of sustainable cooperatives. There are components of cooperative interaction strategies such as data and information flows. The interaction strategy has a major impact on cooperative performance so that this aspect is highly emphasized. There is regular cooperative interaction on priority institutional issues (e.g., strengthening and enhancing cooperative capacity, human resource development, etc.). This aspect can be marked with certain limitations. Data and information usually flow only from the entity (source) to the central level. An informal cooperative interaction strategy exists, but it is not operationalized. Cooperative strategic interactions cover healthy lifestyles and prevention issues. Formal cooperative interaction strategies are available with messages targeted to specific audiences. There is a cooperative interaction strategy with defined messages tailored to specific audiences and purposes informed by institutional evidence. Institutional authorities (government) can measure the impact of strategic interactions and adjust interaction strategies accordingly. Strategic interactions informed by advanced analytics shortly.

Aspects of social participation is an interesting topic as stated in the paper by Fu et al. (41); social participation, social support, instrumental activity, frailty, and loneliness have become research frontiers over the past 5 years. Furthermore, the mention of the health sector has been corroborated by papers from (42), social participation experiences studies yielded positive outcomes regarding health status and quality of life in the communities in which such experiences were implemented. It is also mentioned that social participation in adults must be adapted to their heterogeneous needs and preferences (43). Social participation also requires the right model as an indicator of community diagnosis (44). In multiple sclerosis patients, it is also emphasized how social participation affects the impact of the disease and is related to depression (45). From the literature, it is clear that social participation is an important aspect of health and well-being throughout life as stated by (46). Social participation is also one of the components to reduce the negative psychosocial effects of home confinement, it is highly recommended to implement a national strategy that focuses on promoting social inclusion through technology-based solutions in the COVID-19 era (47). Appropriate social participation for increased attention in routine care, both as a burden trigger and as an outcome of therapy (48). Rigorous studies are needed to evaluate the long-term effect of technology on the multidimensional concept of social participation (49). Transparency and interaction at an early stage can build trust in the system and facilitate contribution and cooperation in various parts of the cooperative. Interaction and engagement with cooperative members and the public through mechanisms to encourage active and transparent decision-making processes within cooperatives. The indicators for this aspect are measured by the interaction between members of the cooperative and the community; One-way interactions (websites, ads, etc.); special activities (campaigns), surveys, forum group discussion, social networks, and website interactions, participation in government bodies, participation in advisory groups, and cooperative members and/or the public are involved directly and indirectly in cooperative decisions. Interaction with cooperative members and the public is usually “one-way” (e.g., through websites and advertisements). Engagement with cooperative members and the public is limited to basic mechanisms such as surveys and focus groups. The participation of cooperative members in the cooperative system is actively encouraged through social media and formal roles in government bodies and advisory groups. Cooperative member institutions and the public are always involved in decisions by transparent institutions, driven by evidence and engagement with cooperative members and the public.

Aspects of the Academic and Scientific Ecosystem make an important contribution to the institution because it has useful values, as stated in the research. A steady reinforced construction of academic scientific communities to improve sustainability (50). Diverse and inclusive scientific communities are more productive, innovative, and impactful (51). According to Martínez-Nicolás (52), a scientific community is committed to the development of the disciplinary field. According to Dibbern and Serafim (53), the academic community, in general, is concerned with the production and dissemination of knowledge, then the academic and scientific community develop tools for managing the infodemic using digital technologies and data science (54). Academic and scientific ecosystems contribute to research and generate new knowledge about cooperatives. In its implementation, it is characterized by several conditions, this institution formally integrates academics in cooperative activities, and this institution has an expert advisory group consisting of selected external experts to advance cooperatives. No formal relationship is established between the cooperative authorities and the academic/scientific ecosystem. Relations with academics are fluid, informal, and on-demand formal relationships with academics have been established to broaden institutional knowledge and learning. Formal links have been established with academia/Scientific Ecosystems focused on supporting projects and programs with specialized studies. Formal links have been established with academia/Scientific Ecosystems focused on supporting a particular project or study, supporting decision-making, and program evaluation.

The last aspect, namely the network, is an important aspect in maintaining the sustainability of the institution, the network has had an impact that is strengthened through research results (55) and an effective approach with the efforts that need to be taken to properly accept and integrate social networks, needs to be taken. Then, research from (56) recommends that the government realize networking governance, in the implementation of Corporate Social Responsibility (CSR) for women's empowerment. Government financial support strongly and strongly supports the relationship between network structure (density and centrality) and sustainable competitive performance (57). The management of product development partnerships in government laboratories has also shown the contribution of the critical success factors model (58). Then, the research by Dauliyeva et al. (59) concludes that the model in offering businesses and governments various ways to build better networks: realizing the benefits of networking in supporting sustainable development, achieving transformation of partnerships to work better for society and the environment; build mutual trust and network responsibility. In line with that, (60) paper also concludes that the government and development partners must carry out monitoring mechanisms to ensure efficient use of borrowed funds (61), collaborative economic development actions that governments are taking, and how this equity can stabilize their local economies. Various types of networks are implemented, such as networks of strategic and diplomatic relations, thematic and knowledge networks, and social networks for the involvement of cooperative members and the community. Conditions that can be indicated are the existence of an internal network to share knowledge, a network between programs to increase the capacity of cooperatives, and participation in an inter-institutional network to share knowledge. Networks for sharing knowledge are usually *ad hoc* and informal. Staff participates in knowledge networks (e.g., ecosystem practices, conferences, and annual member meetings) on an *ad hoc* basis Participation in Ecosystem practices is encouraged and staff regularly capture and share knowledge from these forums. Knowledge networks are integrated into cooperative institutional structures and practices through resource and compensation programs. As an integrated cooperative institutional practice, participating and creating networks is focused on helping cooperatives continuously identify and adapt emerging knowledge.

These six aspects form the basis for efforts to encourage sustainable and sustainable cooperatives by paying attention to pandemic conditions, guided by health protocols, which can be developed in the form of web-based systems and mobile applications to make it easier for cooperative members, cooperatives, policymakers, and the private sector to interact to encourage the acceleration of NER. Web-based systems and mobile applications provide dynamic and informative health records of cooperative members in anticipation of a pandemic. Efforts to develop a web-based system and cooperative mobile application based on health information, the economy of cooperative members, and the development of cooperatives can be carried out with further research due to the limitations of this paper.

## Conclusion

Part of KM and sharing consisting of KP aspects, knowledge design aspects, strategic interaction aspects, social participation aspects, academic and scientific ecosystem aspects, and network aspects can be aspects that support NER's efforts in Indonesia. The indications required in each of the aspects discussed are summarized as a useful alternative section for improving decision-making, capturing, sharing, and measuring institutional knowledge for the success of the NER Program. These six aspects can also be part of a system-based learning organizational framework with information technology interventions in sustainable cooperatives and health. To function effectively in this rapidly changing business environment, the existing situation of organizations is lacking in terms of financial assistance and availability of skilled labor. The challenges identified as the cause/causal sets are crucial in building the resilience of cooperatives. The implications of this research are theoretically building a new framework for economic recovery in every province in Indonesia to be more effective, while practically it can be used as input for making economic recovery strategy decisions in the post-COVID-19 pandemic.

## Data availability statement

The raw data supporting the conclusions of this article will be made available by the authors, without undue reservation.

## Author contributions

All authors listed have made a substantial, direct, and intellectual contribution to the work and approved it for publication.

## Conflict of interest

The authors declare that the research was conducted in the absence of any commercial or financial relationships that could be construed as a potential conflict of interest.

## Publisher's note

All claims expressed in this article are solely those of the authors and do not necessarily represent those of their affiliated organizations, or those of the publisher, the editors and the reviewers. Any product that may be evaluated in this article, or claim that may be made by its manufacturer, is not guaranteed or endorsed by the publisher.
